# Phosphatidylserine-Gold Nanoparticles (PS-AuNP) Induce Prostate and Breast Cancer Cell Apoptosis

**DOI:** 10.3390/pharmaceutics13071094

**Published:** 2021-07-17

**Authors:** Allan Radaic, Nam E. Joo, Soo-Hwan Jeong, Seong-II Yoo, Nicholas Kotov, Yvonne L. Kapila

**Affiliations:** 1 Orofacial Sciences Department, School of Dentistry, University of California, San Francisco (UCSF), San Francisco, CA 94143, USA; allan.radaic@ucsf.edu (A.R.); nejoo9136@gmail.com (N.E.J.); 2 Department of Chemical Engineering, Kyungpook National University, Daegu 41566, Korea; shjeong@knu.ac.kr; 3 Department of Polymer Engineering, Pukyong National University, Busan 608737, Korea; siyoo@pknu.ac.kr; 4 Department of Chemical Engineering, College of Engineering, University of Michigan, Ann Arbor, MI 48109, USA; kotov@umich.edu

**Keywords:** gold nanoparticles, phosphatidylserine, PS-AuNP, breast cancer, prostate cancer, apoptosis

## Abstract

Prostate and breast cancer are the current leading causes of new cancer cases in males and females, respectively. Phosphatidylserine (PS) is an essential lipid that mediates macrophage efferocytosis and is dysregulated in tumors. Therefore, developing therapies that selectively restore PS may be a potential therapeutic approach for carcinogenesis. Among the nanomedicine strategies for delivering PS, biocompatible gold nanoparticles (AuNPs) have an extensive track record in biomedical applications. In this study, we synthesized biomimetic phosphatidylserine-caped gold nanoparticles (PS-AuNPs) and tested their anticancer potential in breast and prostate cancer cells in vitro. We found that both cell lines exhibited changes in cell morphology indicative of apoptosis. After evaluating for histone-associated DNA fragments, a hallmark of apoptosis, we found significant increases in DNA fragmentation upon PS-AuNP treatment compared to the control treatment. These findings demonstrate the use of phosphatidylserine coupled with gold nanoparticles as a potential treatment for prostate and breast cancer. To the best of our knowledge, this is the first time that a phosphatidylserine-capped AuNP has been examined for its therapeutic potential in cancer therapy.

## 1. Introduction

Phosphatidylserine (PS) is an essential lipid in eukaryotic cellular bilayer lipid membranes, and therefore has structural and biochemical importance [1,2]. It is the most abundant negatively charged glycerophospholipid in cell membranes, where it is actively maintained on the membrane’s inner leaflet by flippase enzymes [1].

PS is widely known for its emblematic participation in apoptosis, in which PS is externalized, upon losing membrane asymmetry. These exposed PS then interact with a set of extracellular serum proteins and PS receptors, triggering an array of biochemical and immunological responses that evoke recognition of the apoptotic bodies by phagocytes, which tag cells with an “eat me” signal for efferocytosis [1,3]. Efferocytosis is the ability of phagocytic cells to ingest, process, and remove apoptotic cells without inducing inflammation, and it is necessary for maintaining homeostasis [4]. However, recent data demonstrate that PS itself may play a more complex role in apoptosis than previously imaged [5], since PS regulates other cellular functions, such as providing an important docking site for several proteins with poly-cationic domains and for membrane-cytoskeletal anchoring, and structurally contributing to the cell membrane curvature and fluidity [1,6].

However, tumors hijack this immune detection via PS exposure by the creation of a local immunosuppressive environment, comprising of IL-10, TGF-β, soluble FAS and FAS-ligand [4], and diverse sources of exposed PS [3]. Thus, new strategies intervening in this PS exposure and hijacking it may be useful to treat cancer.

Nanotechnology is an approach used to produce and study materials in the nanoscale size range [7,8]. Nano-sized materials have unique features compared with their bulk counterparts that are being used to improve therapeutic agents and have led to the creation of the field of nanomedicine [7,8]. Nanomedicine has sought to improve the use of low-weight molecular agents, such as lipids, proteins, and genes to treat diseases and these have been highlighted in the literature as promising tools for expanding current therapies, including cancer therapy [9,10,11,12,13]. Among these strategies, gold nanoparticles (AuNPs) have been well documented in the literature due to their large surface area, biocompatibility [14,15,16,17,18,19], unique biomimetic [20], and optical and drug delivery properties [21]. Biomimetic AuNP technology has shown promising results in terms of safety and efficacy in delivering cytotoxic agents in vivo [22] and in ongoing clinical trials [23,24]. Yet, as of 2016, no FDA-approved AuNP have been reported [25]. In addition, a significant advantage of AuNP is their potential for modifying the surface of a particle with different targeted and functionalized agents, which significantly broadens the range of AuNP biomedical applications, particularly for cancer treatment [18]. Sztandera et al. [18] argue that functionalized AuNP exhibit good biocompatibility and controllable biodistribution patterns, making AuNP promising candidates for innovative therapies. For instance, functionalizing AuNP would make them very suitable for use as drug delivery agents that target cancer cells [22] or in cancer gene therapy [15].

So far, very little has been done with phosphatidylserine-caped gold nanoparticle (PS-AuNP) in the literature, including a copper detector for river water [26], daunomycin aptamer [27], and a detector of virus-containing compartments in macrophages [28]. To the best of our knowledge, none of these formulations were used as nanomedicine, especially for cancer therapy.

Therefore, in this study, we synthesized a PS-AuNP formulation and investigated its potential to promote apoptosis in breast and prostate cancer cells.

## 2. Materials and Methods

### 2.1. PS-AuNP Synthesis

**Figure 1:**
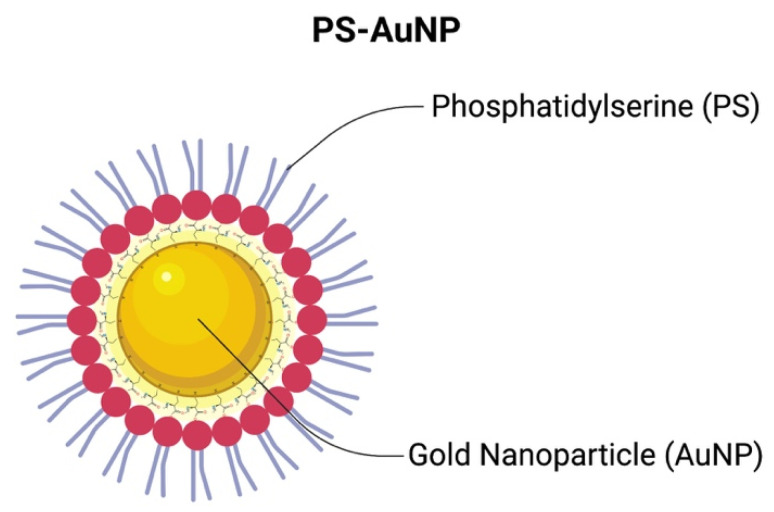
Schematic structure of phosphatidylserine-capped gold nanoparticle (PS-AuNP) after EDC/NHS coupling.

AuNP were synthesized using the Turkevich method [29]. Briefly, chloroauric acid (HAuCl_4_) solution was heated until boiled. Then, under vigorous stirring, a 1% sodium citrate solution was added to form the citrate-stabilized AuNPs. Then, it was stirred for 20 min under heat and allowed to cool down. To form PS-AuNP, the AuNP solution underwent a place-exchange reaction with L-cysteine for 30 min to provide thiol linkages on the AuNP surface and reactive carboxylic groups on the terminal end imparting chirality to the NP, which is essential for nanomedicine. Next, the l-cysteine-AuNP solution was centrifuged at 4000× *g* to separate the nanoparticles from the non-bound l-cysteine, and the l-cysteine-AuNP were dissolved in water. Then, PS was linked to the l-cysteine-AuNP by 1-ethyl-3-(3-dimethylaminopropyl) carbodiimide hydrochloride (EDC) and *N*-hydroxysulfosuccinimide (Sulfo-NHS) coupling. Briefly, EDC and NHS react with l-cysteine, forming a amine-reactive NHS-ester. This reactive ester then reacts with the primary amine found at the hydrophilic head of PS, establishing a stable amide bond between PS and AuNP, and, thus, forming PS-AuNP. Finally, the solution was centrifuged at 4000× *g* to remove any reaction leftover, and PS-AuNP was then dispersed in water and stored in 4 °C until further use. Through this coupling method, we expect that the EDC/NHS links the head of the phospholipid to the nanoparticle, thus functionalizing the nanoparticle (PS-AuNP), as in Figure 1.

### 2.2. PS-AuNP Characterization

AuNP with and without PS were characterized by UV-vis Spectroscopy with the use of the AuNP surface plasmon resonance (SPR) effect using a Spectramax M2 microplate reader (Molecular Devices, San Jose, CA, USA). Haiss et al. [30] proposed that, for particles with diameter lower than 35 nm, the following ratio (R) is proportional to the diameter of the gold nanoparticle:

(1) $$d_{AuNP}\;\sim \;R=\;\frac{A_{SPR}}{A_{450}}$$

Here, *A_SPR_* is the samples absorbance at the SPR effect peak, and *A_450_* is the sample absorbance at 450 nm. A table containing the correlation between *R* and the actual AuNP diameters can be found in Haiss et al. [30].

### 2.3. Cell Culture

The prostate cancer cell lines LNCaP and PC3 were maintained in RPMI-1640 medium supplemented with 10% Fetal Bovine Serum (FBS) (Gibco, Waltham, MA, USA) and 1% penicillin/streptomycin (Pen/Strep) (Thermo-Fisher, Waltham, MA, USA).

The breast adenocarcinoma cell lines MDA-MB-231, CAL-51, HS578-T, and MCF-7; the prostate cancer cell line DU-145, and the oral cancer cell lines HSC-3, UM-SCC-11A and UM-SCC-17B were maintained in DMEM medium supplemented with 10% FBS (Gibco, Waltham, MA, USA) and 1% Pen/Strep (Thermo-Fisher, Waltham, MA, USA).

The normal human breast epithelial fibroblast MCF-10A and the prostate cancer cell line C4-2b were maintained in DMEM/F-12 medium supplemented with 10% Fetal Bovine Serum (FBS) (Gibco, Waltham, MA, USA) and 1% penicillin/streptomycin (Pen/Strep) (Thermo-Fisher, Waltham, MA, USA).

The normal human prostate epithelial (HPrEC) cell line (Lifeline Cell Technology, Carlsbad, CA, USA) was maintained in ProstaLife^TM^ prostate epithelial cell culture medium (Lifeline Cell Technology, Carlsbad, CA, USA) supplemented with Prostalife^TM^ LifeFactors (which includes transforming growth factor-α, epinephrine, insulin, transferrin, and hydrocortisone).

The normal human gingival keratinocytes (GKT) cell line was maintained in Dermal Cell Basal Medium (ATCC, Manassas, VA, USA) supplemented with Keratinocyte Growth Kit (ATCC, Manassas, VA, USA).

All cells were maintained under a humid atmosphere at 37 °C and 5% CO_2_ and subcultured every 2 or 3 days using trypsin/PBS (Gibco, Waltham, MA, USA).

### 2.4. Cell Morphology under Light Microscopy

All cell lines were seeded in 96-well plates at 1 × 10^4^ cells/well and were allowed to adhere overnight. Then, all 4 cell lines were challenged with PBS (control), or 150 nM of either AuNP or PS-AuNP for 72 h and their morphology was captured using EVOS^tm^ XL Core light microscopy (Thermo-Fisher Scientific, Waltham, MA, USA).

### 2.5. Cell Morphology Quantification Analysis

All captured images were subjected to a semi-quantification analysis using FIJI/ImageJ software. Briefly, each cell in the obtained picture was individualized and their area and perimeter were obtained. With this information, the cell circularity was calculated by the following formula:

$$Circularity=4\pi (A/P^{2})$$

Here, *A* is the individual cell’s area, and *P* is its perimeter. A circularity value of 1.0 indicates a perfect circle, while values close to 0 indicate an elongated polygon, such as a rectangle or a diamond shape.

### 2.6. Scanning Electron Microscopy

MDA-MB-231 cells were seeded in 24-wells plates containing sterilized coverslips at 5 × 10^5^ cells/well and were allowed to adhere overnight. Then, the cells were treated with PBS (control), or 150 nM of either AuNP or PS-AuNP for 72 h. Next, the cells were fixed with 2.5% glutaraldehyde in PBS at 4 °C overnight and serial dehydrated using different solutions of ethanol (50%, 60% 80%, 90%, 95%, and 100%), for 15 min, each. Finally, cells were sputtered and visualized with a Scanning Electron Microscope.

### 2.7. DNA Fragmentation

Cell apoptosis was evaluated by the level of histone-associated DNA fragments (mono- and oligonucleosomes) using the Cell Death Detection ELISA^PLUS^ kit (Sigma-Aldrich, St. Louis, MO, USA), according to the manufacturer’s instructions. Briefly, cell lysates were placed in a streptavidin-coated microplate and incubated with a mixture of anti-histone-biotin and anti-DNA peroxidase. Then, the optical density (405 nm) of the samples was measured on a Spectramax M2 microplate reader (Molecular Devices, San Jose, CA, USA). According to the kit’s manual, 1 × 10^−3^ OD is equivalent to 1mU of Histone/DNA fragments.

### 2.8. Statistical Analysis

Data were analyzed by a two-way ANOVA. Intergroup differences were analyzed by Tukey’s post hoc test and *p* < 0.05 was considered significant. All experiments were performed at least three times and in triplicate and results are presented as mean ± SD.

## 3. Results

**Figure 2:**
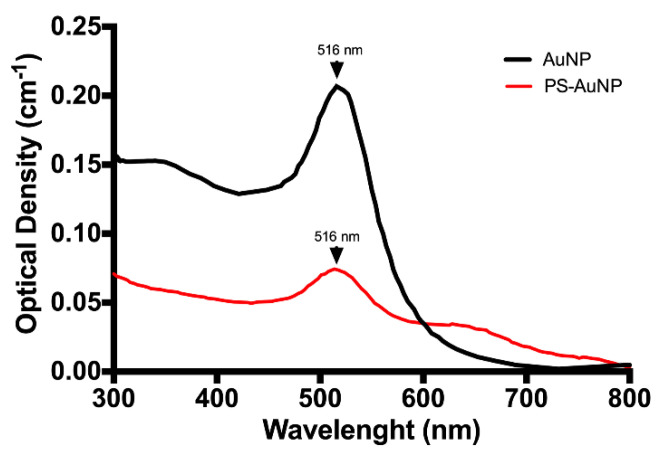
AuNP (Black line) and PS-AuNPs (Red line) characterization via surface plasmon resonance (SPR) effect.

Citrate-stabilized AuNP has been extensively characterized in literature [31,32,33,34,35]. In addition, the TEM of this particular AuNP was previously published by Kim et al. [36]. Thus, AuNP with and without PS were characterized by surface plasmon resonance (SPR) effects, as described by Haiss et al. [30] and Agarwal et al. [37]. After synthetization, the AuNP showed an SPR peak at 516 nm and a ratio A_SPR_ to A_450_ of 1.561, which indicates that the synthesized AuNP have a diameter of 12 nm. Then, phosphatidylserine (PS) was linked to AuNP via EDC/NHS coupling, after substituting the stabilizing agent (from citrate to l-cysteine). After coupling, the PS-AuNP were characterized similar to the AuNP (Figure 2—red line). We found a red-shift for the PS-AuNP spectra compared to the AuNP, with a decrease in the SPR peak (at 516 nm) and the formation of a broad band between 600–700 nm, indicating an increase in the nanoparticle diameter > 100 nm, possibly due to PS incorporation and/or aggregation. Then, with these numbers, we quantified the Au concentration and the number of particles in each solution. In our case, we found an Au concentration of 0.55 mM and the equivalent of 1.1 × 10^10^ nanoparticles/mL for AuNP, while PS-AuNP had an Au concentration of 0.21 mM and 0.42 × 10^10^ nanoparticles/mL. This difference may be due to losses during the PS coupling process.

**Figure 3:**
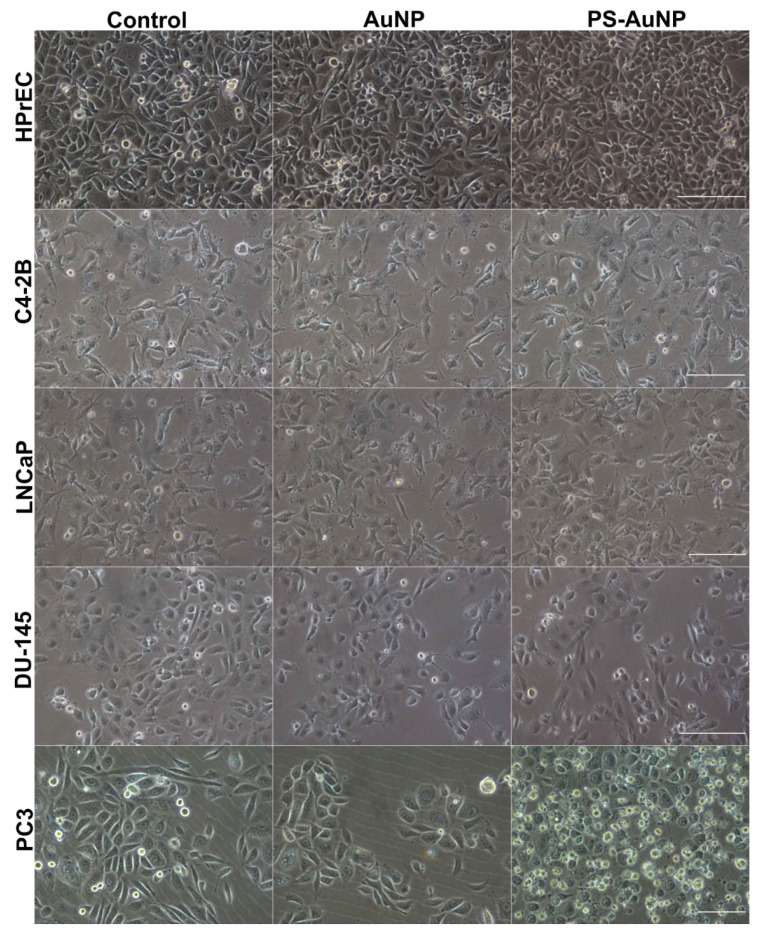
PS-AuNPs induce significant morphological changes in prostate cancer (PC3) cells compared to PBS-challenged cells (control) and the normal prostate (HPrEC) cell line. From top to bottom rows, light microscopy of the cell morphology of HPrEC normal prostate cell line and the prostate cancer cell lines C4-2B, LNCaP, DU-145, and PC3. The left column shows cell lines challenged with PBS; the central column shows cell lines challenged with AuNP; and the right column shows cell lines challenged with PS-AuNP. Scalebar equivalent to 50 µm.

**Figure 4:**
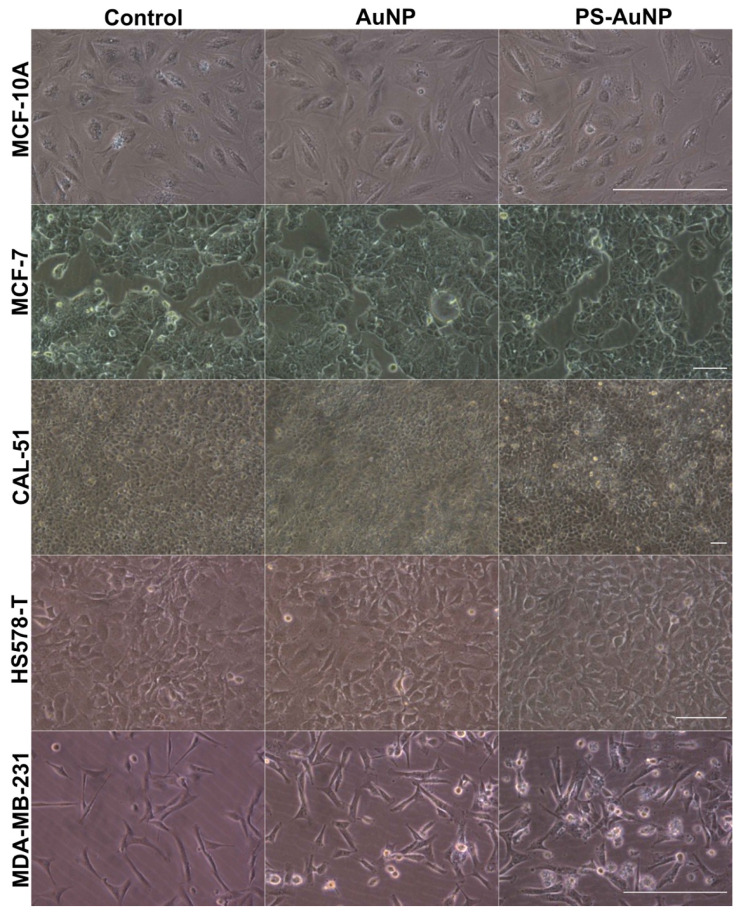
PS-AuNPs induces significant morphological changes in breast cancer (MDA-MB-231) cells compared to PBS-challenged cells (control) and the normal breast (MCF-10A) cell line. From top to bottom rows, light microscopy of the cell morphology of MCF-10A normal breast cell line and the breast cancer cell lines MCF-7, CAL-51, HS578-T, and MDA-MB-231. The left column shows cell lines challenged with PBS; the central column shows cell lines challenged with AuNP; and the right column shows cell lines challenged with PS-AuNP. Scalebar equivalent to 50 µm.

**Figure 5:**
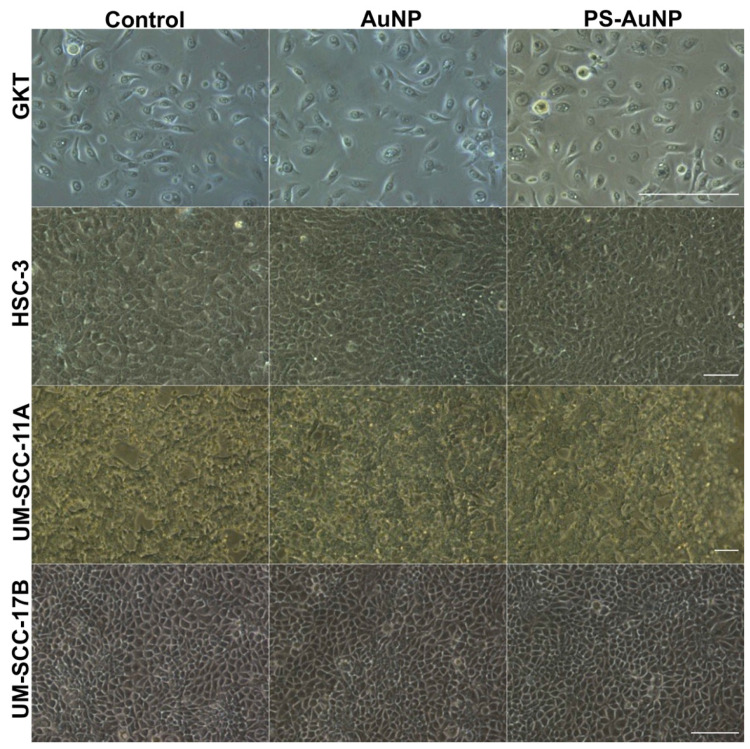
PS-AuNPs do not induce significant morphological changes in oral cancer cells. From top to bottom rows, light microscopy of the cell morphology of GKT normal gingival keratinocyte cell line and the oral cancer cell lines HSC-3, UM-SCC-11A, and UM-SCC-17B. The left column shows cell lines challenged with PBS; the central column shows cell lines challenged with AuNP; and the right column shows cell lines challenged with PS-AuNP. Scalebar equivalent to 50 µm.

Next, PS-AuNP were screened for their ability to promote morphological changes in a panel of several prostate (Figure 3), breast (Figure 4), and oral (Figure 5) cancer cell lines compared to their normal counterpart cell lines. PS-AuNP treatment triggered pronounced morphological effects in PC3 and MDA-MB-231, as they lost their spindle shape compared to the controls, and exhibited cell shrinking, irregular shapes, and their nuclei were no longer discernable, whereas treatment with the AuNP alone appeared to have small/minor effects on cell morphology—increased rounding of the cells. Interestingly, no significant morphological changes were observed on the rest of the cancer cell lines tested. This differential response among cell lines may be due to heterogeneity among cell lines [38].

Remarkably, both AuNP and PS-AuNP did not induce any morphological changes in both normal prostate (HPrEC), breast (MCF-10A), and oral (GKT) cell lines, indicating specificity of the nanoparticles, specifically PS-AuNP against the aggressive and metastatic MDA-MB-231 (breast) and PC3 (prostate) cancer cell lines.

**Figure 6:**
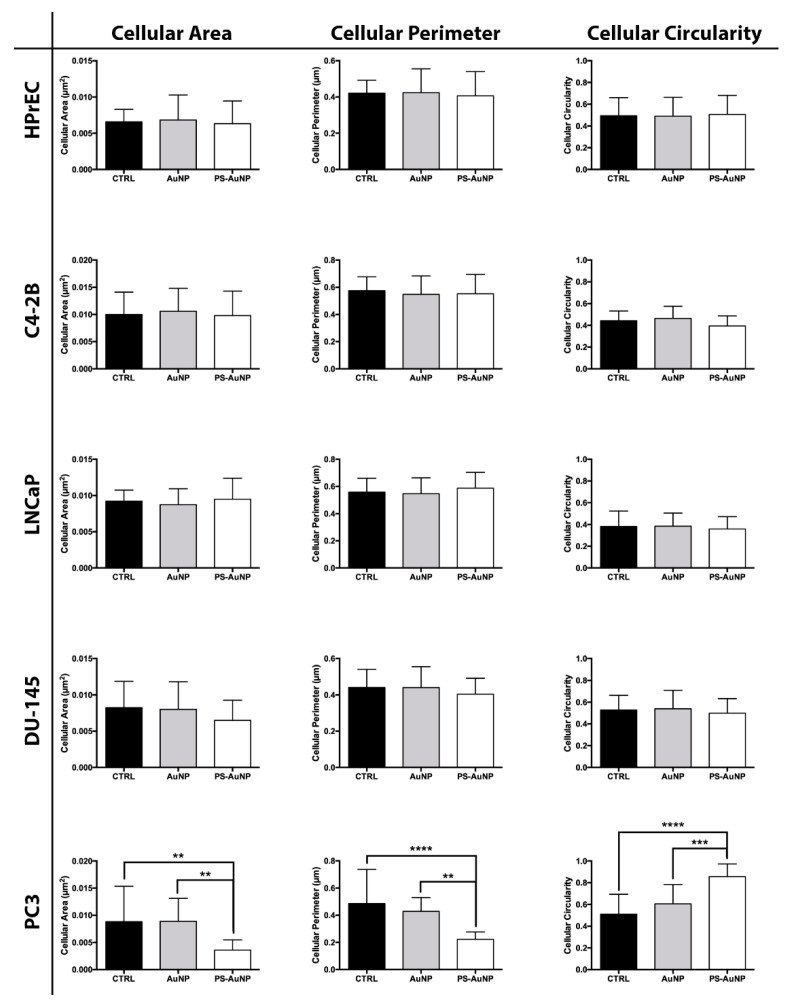
PS-AuNPs induce significant decreases in cellular area and perimeter and significant increases in cell circularity in prostate cancer (PC3) cells, compared to PBS (control) and AuNP-challenged cells, but not in normal prostate (HPrEC) cell line. Cellular Area (left), Cellular Perimeter (Center), and Cell Circularity (Right) of the HPrEC normal prostate cell line and the prostate cancer cell lines C4-2B, LNCaP, DU-145, and PC3. ** means *p* < 0.01 between the annotated samples; *** means *p* < 0.001 between the annotated samples; and **** means *p* < 0.0001 between the annotated samples.

**Figure 7:**
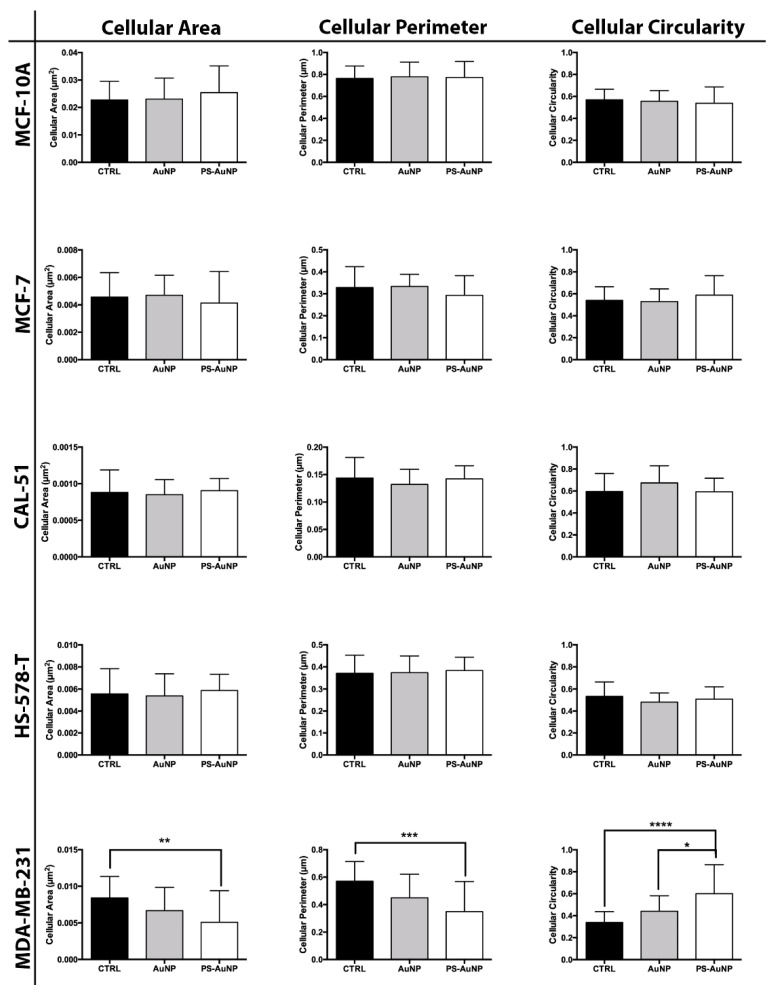
PS-AuNPs induce significant decreases in cellular area and perimeter and significant increases in cell circularity in breast cancer (MDA-MB-231) cells compared to PBS (control) and AuNP-challenged cells, but not in a normal prostate (MCF-10A) cell line. Cellular Area (left), Cellular Perimeter (Center), and Cell Circularity (Right) of MCF-10A normal prostate cell line and the prostate cancer cell lines MCF-7, CAL-51, HS578-T, and MDA-MB-231. * means *p* < 0.05 between the annotated samples; ** means *p* < 0.01 between the annotated samples; *** means *p* < 0.001 between the annotated samples; and **** means *p* < 0.0001 between the annotated samples.

**Figure 8:**
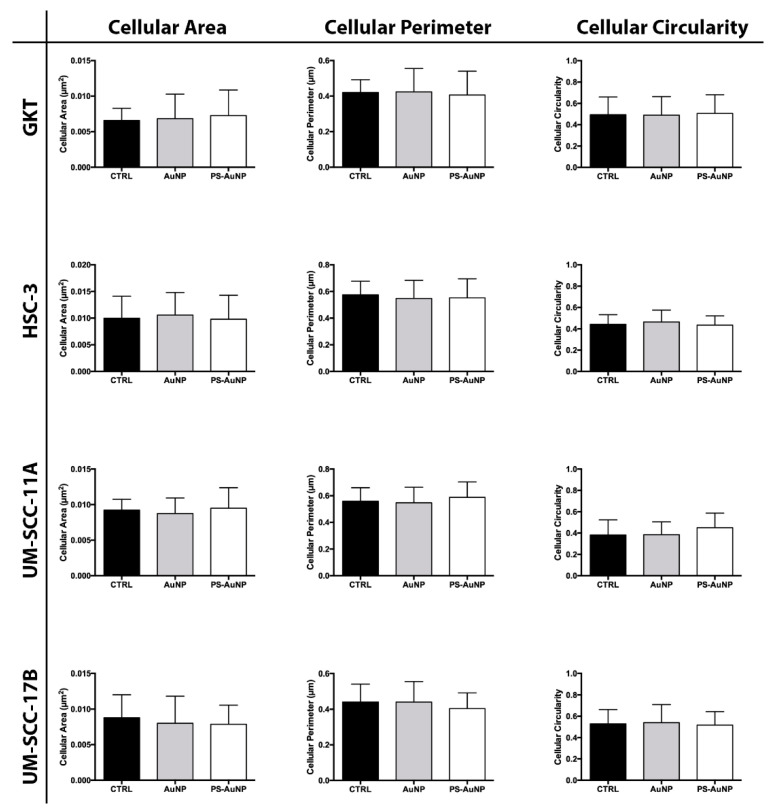
PS-AuNPs do not induce significant changes in cellular area, perimeter, and circularity in either a normal oral (GKT) cell line or in all oral cancer cell lines. Cellular Area (left), Cellular Perimeter (Center), and Cell Circularity (Right) of GKT normal prostate cell line and the prostate cancer cell lines HSC-3, UM-SCC-11A, and UM-SCC-17B.

To validate the observed morphological changes, we further analyzed and quantified the obtained images for average cellular area, average cellular perimeter, and the circularity of the prostate (Figure 6), breast (Figure 7), and oral cancer (Figure 8) cells. We found significant decreases in the cellular area and perimeter, as well as significant increases in cellular circularity for PS-AuNP-treated cells compared to controls in the MBA-MD-231 and PC3 cell lines, while no significant differences were found between AuNP and the control-treated cells. Interestingly, we found a significant increase in cellular circularity for PS-AuNP-treated cells compared to AuNP-treated cells for both PC3 and MBA-MD-231 cell lines, and a significant decrease in cellular area and perimeter for PS-AuNP-treated PC3 cells, indicating that the incorporation of PS may be responsible for these effects.

**Figure 9:**
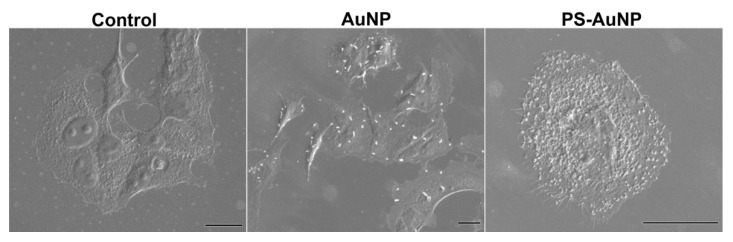
PS-AuNPs induce morphological changes consistent with apoptosis in MDA-MB-231 cells. Scanning electron microscopy of MDA-MB-213 cells challenged with either PBS (control—left panel), AuNP (central panel), or PS-AuNP (Right Panel). Scale bar equivalent to 20 µm.

To further analyze the PS-AuNP effects on the MDA-MB-231 cell line, we treated the cells with either AuNP or PS-AuNP and imaged the results using scanning electron microscopy (SEM) (Figure 9). Compared to the PBS control, AuNP treatment did promote intrinsic toxicity in the cells, although there were some changes in cell shape, and the AuNP were scattered throughout the cell. However, PS-AuNPs challenged cells exhibited significant changes in cell shape as they shrunk, were condensed, and had a rounded shape, consistent with apoptosis. Interestingly, PS-AuNP seem to promote membrane ruffling in these cells, further indicating a potential induction of apoptosis.

Thus, we hypothesized that PS-AuNP induces apoptosis in both prostate and breast cancer cell lines.

**Figure 10:**
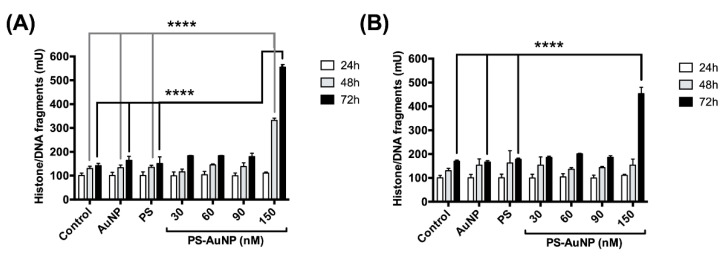
PS-AuNPs induce the formation of Histone/DNA fragments in PC3 (**A**) and MDA-MB-231 (**B**) cell lines. PC3 and MDA-MB-231 cells were challenged for 24 h, 48 h, or 72 h with either PBS (control), 150 nM of AuNP (gold only), 150 nM of PS (lipid only), or a range of PS-AuNP concentrations (from 30 to 150 nM) and tested for the amount of histone/DNA fragments after challenge. **** means *p* < 0.0001 between the annotated samples.

Therefore, we tested this hypothesis by quantifying the total amount of histone-associated DNA fragments in these cells after challenging them with either 150 nM of AuNP (gold only), 150 nM of PS (lipid only), and a range of PS-AuNP concentrations for 24 h, 48 h, or 72 h (Figure 10). No significant increase in DNA fragmentation was found for either AuNP or PS up to 72 h, compared to the control. However, for PS-AuNP, 150 nM triggered significant DNA fragmentation at both 48 h and 72 h for PC3 cells and only after 72 h for MDA-MB-231 cells.

**Figure A1:**
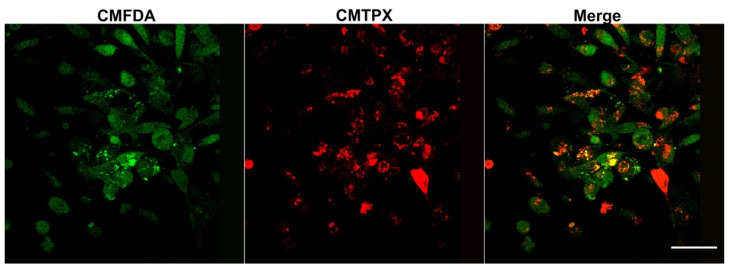
—MCF-10A cells engulf MDA-MB-231 cells after both are treated with PS-AuNP. MCF-10A and MDA-MB-231 cells were stained with Cell Tracker Green (CMFDA) and Cell Tracker Red (CMTPX), respectively. The cells were then trypsinized, co-seeded in a glass bottom 4-well chamber, and allowed to adhere overnight. Finally, the cells were challenged with 150 nM of PS-AuNP for 72 h and imaged.

Remarkably, preliminary results (Figure A1) show that co-culturing CMFDA-labeled MCF-10A with CMTPX-labelled MDA-MB-231 and challenging them with PS-AuNP triggers MCF-10 to actively phagocytose MDA-MB-231 cells. This suggests that PS-AuNP mediates cancer cell apoptosis that is recognized by normal phagocytic cells that then remove the apoptotic cells by efferocytosis.

## 4. Discussion

Breast, prostate, and oral cancers are the current first, second, and eighth leading causes of new cancer cases [39]. Phosphatidylserine (PS) is an essential lipid in the cellular bilayer lipid membrane. It is usually present in the inner leaflet of the cellular membrane, and its exposure in the outer leaflet triggers macrophage efferocytosis [1,4]. However, PS signalizing is dysregulated in tumors, antagonizing the immune response [1]. Therefore, in this study, we synthesized a phosphatidylserine-capped gold nanoparticle (PS-AuNP) formulation and investigated its ability to induce apoptosis in breast, prostate, and oral cancer cells compared to normal control cells.

Initially, l-cysteine was adsorbed onto Au nanoparticles via a thiol-gold interaction. Then, NHS/EDC was used to couple l-cysteine carboxylic acid to the primary amine at the PS polar head via a stable amine bond. With this configuration, we expect that the PS carbon-chain would be exposed, making it possible to dock onto the cell’s membrane. Due to the presence of PS, we expect the ζ-Potential to be highly negative for this formulation, similar to PS micelles and liposomes [40].

Our results show that PS-AuNP induces morphological changes and histone/DNA fragmentation compatible with apoptosis only in the metastatic cell lines PC3 and MDA-MB-231, but not in other prostate and breast cancer cell lines. Prostate cancer cell lines are heterogenous and this may account for their differential responses to treatments [38]. For instance, Lima et al. [41] differentiated five prostate cancer cell lines based on their metabolomic profile, including PC3, DU-145, and LNCaP cells, using gas chromatography-mass spectrometry (GC-MS). In analyzing the data, the authors found different alcohol profiles between the cells; these could be used for the differential synthesis of cellular membrane precursors, which could lead to very different cellular membrane profiles between the cells. In addition, these prostate cancer cell lines have differences in terms of their receptor expression, prostate-specific antigen expression, and metastatic status. For instance, LNCaP cells express androgen receptors and prostate-specific antigen, and are considered to have low metastatic potential. DU-145 and PC3 cells, on the other hand, do not express androgen receptors and prostate-specific antigen, and are considered to have moderate and high metastatic potential, respectively [41,42,43,44]. Interestingly, Guo et al. [45] showed that different apoptosis mechanisms were activated when these three cell lines were exposed to the same treatment. The authors showed that, upon treatment with a Protein Kinase C (PKC) inhibitor, DU-145 cells underwent apoptosis mediated by the activation of JNK1, resulting in Caspase-8 cleavage and Cytochrome C release to the cytoplasm. On the other hand, the same treatment led to LNCaP and PC3 apoptosis via Reactive Oxygen Species (ROS), mediated by an unfolded protein response and GADD153. Taken in aggregate, these data indicate that different responses among prostate cell lines can be expected, due to their different molecular profiles.

Similarly, breast cancer cell lines are very heterogeneous and defined in part by differences in their mutational status in the breast cancer tumor suppression gene (BRCA1), estrogen receptor (ER), progesterone receptor (PR), and human epithelial receptor 2 (HER2) [46]. Dai et al. [46] recently evaluated the morphological, molecular (including mRNA and protein), and mutational differences of 84 breast cancer cell lines and were able to categorize them into five different groups, consistent with the actual breast cancer classification. Using their comparative analyses, we were able to identify very different molecular profiles among the chosen cell lines in this work. For instance, MCF-7 is classified as an invasive ductal carcinoma (IDC) and is positive for ER and PR. HS-578-T is also classified as an IDC, but it is negative for all the receptors (triple negative). MDA-MB-231 is classified as an adenocarcinoma and is negative for all the receptors too (triple negative). Additionally, He et al. [47] found significantly different membrane profiles in seven breast cancer cell lines, including MDA-MB-321 and MCF-7 cells. These different membrane profiles may result in a distinct ζ-Potential among the different cell lines [40], which may enable the attraction of differential amounts of the PS-AuNP to the different cell types. Thus, these differences may also account for the differential response to PS-AuNP by different cancer cell lines. Thus, our results underscore the importance of screening several different cell lines within a cancer type, as their mutational status, receptor expression, aggressivity, and membrane potential/profiles may play a significant role in the efficacy of nanomedicines, as demonstrated by this study.

Interestingly, our data also showed no major effects on oral cancer cells following treatment with the PS-AuNP. Liu et al. [48] recently reported increased PS in blood cells, microparticles, and serum-cultured endothelial cells in patients with oral squamous cell carcinoma (OSCC) compared to healthy controls. This could indicate that OSCC may use PS over-expression as an immunosuppressive strategy for tumor progression, especially for stage III/IV cancers. Further, this suggests that PS blockade may be a viable therapeutic strategy for treating such patients. In addition, Abboud-Jarrous et al. [49] showed that Proteins S (PROS1), a PS receptor [50], mediates OSCC survival, proliferation, and migration through regulation of AXL, a proto-oncogenic receptor protein. These finding suggest that PROS1 may be involved in OSCC tumorigenesis and immunosuppression and may be a potential novel OSCC therapeutic target. Thus, PS-AuNP may not mediate apoptotic effects in OSCC cells because of this PS survival mechanism already in place in OSCC cells.

May et al. [51] recently demonstrated that AuNP induces DNA fragmentation in a lung cancer (A594) cell line via reactive oxygen species (ROS) after 24 h. Interestingly, most of these damages were largely repaired 72 h after treatment initiation by the cells and a minor growth lag was found after six days of treatment compared to control cells. Our results, on the other hand, demonstrate that capping AuNP with PS, significantly enhances AuNP’s DNA fragmentation ability in both prostate and breast cancer cells, especially after 72 h. Further, our results demonstrate that these same PS-AuNP do not compromise normal breast and prostate cells.

For many years, PS externalization was merely an “eat me” signal [1,3]. However, more recent data demonstrates that PS itself may play a role in apoptosis and regulate other cellular functions. For instance, Sommet et al. [5] demonstrated that PS exposure is necessary for ADAM17, a prominent protein of the “disintegrin and metalloproteinase” (ADAM) family, to exert its sheddase activity, cleaving transmembrane substrates during apoptosis. In this context, it is possible that the delivery of PS via AuNP may induce ADAM17 sheddase activity, possibly leading to cell shrinkage and apoptosis, as seen in both prostate and breast cancer cells. In contrast, normal prostate and breast cell lines are not affected by the same process. Further studies are needed to determine the mechanism by which PS-AuNP induces apoptosis in cancer cells.

The SEM results show both AuNP and PS-AuNP particles inside or on the top of the cells. Thus, further studies are needed to evaluate whether the nanoparticles are indeed internalized by the cancer cells or attached to the cell membrane. This may be an important step in understanding the mechanism by which PS-AuNP induces apoptosis. One way to check the fate of the nanoparticles in the cell is by fluorescently labelling the nanoparticle and visualizing its distribution using Confocal Laser Scanning Microscopy (CLSM) [52,53]. Du et al. [52] determined the cellular distribution of Polyamine-modified AuNP using this technique and found that the nanoparticles were only attached to the cell membrane and not internalized. Several molecular dynamic simulations demonstrated that anionic lipid-coated AuNP bind to model bilayers via electrostatic interactions with zwitterionic lipids of the membrane (e.g., dipalmitoyl-phosphatidylcholine) [54,55,56,57]. Interestingly, Simonelli et al. [55] demonstrated that anionic lipid-coated AuNP penetrate deep into the membrane bilayer via a three-step process. Initially, electrostatic interactions between the anionic nanoparticle and the zwitterionic lipids in the membrane promote membrane surface adhesion of the nanoparticle. Then, hydrophobic forces pull the nanoparticle deeper into the membrane. Lastly, charged ligands anchored to both membrane leaflets embed the nanoparticles in the membrane core. The authors report that this anchoring is highly favorable and not reversable via free energy. In contrast, Kang and Ko [58] tested the fate of an AuNP coated with an anionic mixture of lipids (dipalmitoyl-phosphatidylcholine, dipalmitoyl-phosphatidylglycerol, cholesterol and distearoyl-phosphatidylethanolamine) and found that the nanoparticles were fully internalized despite being attached to the cell membrane initially. In case the PS-AuNP are internalized, the cellular process by which they are internalized may depend on the specific endocytic pathway utilized by the particular cell line, as molecular dynamic simulations have demonstrated a significant energy barrier for anionic lipid-coated AuNP to fully penetrate the cell by trespassing the membrane bilayer [59]. Comprehensive reviews of cellular mechanisms for nanoparticle internalization and strategies on how to study nanoparticles and biological system interactions have recently been reported [10,53,60]. The differential response of cancer cells to PS-AuNPs compared to normal cells may depend on structural differences in membrane composition between cancer cells versus normal cells; this remains to be elucidated [61,62].

Some epithelial cells [63], including mammary epithelial cells [64], are known to engulf apoptotic cells via efferocytosis by using the same receptors used by macrophages, such as phosphatidylserine receptors (PSR). In the breast tissue, epithelial cell efferocytosis is an important step during mammary gland involution, in which milk-producing mammary epithelial cells return to a near pre-pregnant state via apoptosis [65,66]. During this process, effective clearance of the dying cells is essential to maintain tissue homeostasis [65]. In this context, our preliminary data may also shed light on a possible efferocytosis clearance mechanism, whereby the MCF-10A cells phagocytose the apoptotic MDA-MB-231 cells after PS-AuNP exposure. However, further studies are warranted to validate this PS-AuNP-mediated apoptosis/phagocytosis mechanism.

The in vivo use of biomimetic AuNP technology has shown promise in terms of the safety and efficacy in delivering cytotoxic agents [22]. Both phosphatidylserine and colloidal gold nanoparticles (AuNP) are considered safe for use in humans. Indeed, colloidal AuNP has been tested in a Phase I clinical trial and this trial found that doses from 50 μg/m^2^ to 600 μg/m^2^ were well tolerated [67]. Biomimetic AuNP has been recently reported in three ongoing clinical trials for lung (NCT01679470), prostate (NCT02680535), and head and neck cancer (NCT00848042) [23]. Remarkably, the recently published prostate clinical trial report found the nanoparticles to be safe for use, with no serious adverse effects or significant changes in genitourinary function [24]. In agreement with these findings, in our study, no morphological changes were found in the different normal cell lines examined. Phosphatidylserine is currently used as a dietary supplement and promoted for its ability to improve cognitive function; thus, interest has developed for its use in treatment of Alzheimer’s disease and attention-deficit hyperactivity disorder. Thus, PS-AuNP may be a useful new therapeutic tool for several applications, including in the treatment of breast and prostate cancers. Additionally, to the best of our knowledge, this is the first time that a phosphatidylserine-capped AuNP has been tested for cancer use. Yet, as of 2016, no FDA approved AuNP was reported [25].

## 5. Conclusions

In this proof-of-concept study, we show that phosphatidylserine-capped AuNP (PS-AuNP) significantly changes the morphology and increases DNA/histone fragments in both prostate (PC3) and breast (MDA-MB-231) cancer cells, compared to the controls, but not in normal prostate and breast cell lines. These results suggest that PS-AuNP induces selective apoptosis in both prostate and breast cancer cell lines, but are safe for normal tissue. Thus, nanomedicine approaches may be useful for creating new potential treatments for breast and prostate cancer, two common human diseases that are associated with high morbidity and mortality.

## Data Availability

Not applicable.
